# Effects of obesity and chronic low back pain on gait

**DOI:** 10.1186/1743-0003-8-55

**Published:** 2011-09-26

**Authors:** Veronica Cimolin, Luca Vismara, Manuela Galli, Fabio Zaina, Stefano Negrini, Paolo Capodaglio

**Affiliations:** 1 Bioengineering Department, Politecnico di Milano, Italy; 2 Laboratorio di Ricerca in Biomeccanica e Riabilitazione, Ospedale San Giuseppe, Istituto Auxologico Italiano, Via Cadorna 90, I-28824, Piancavallo (VB), Italy; 3 IRCCS "San Raffaele Pisana" Tosinvest Sanità, Roma, Italy; 4 Italian Scientific Spine Institute (ISICO), Via Bellarmino 13/1, Milan, Italy

## Abstract

**Background:**

Obesity is often associated with low back pain (LBP). Despite empirical evidence that LBP induces gait abnormalities, there is a lack of quantitative analysis of the combined effect of obesity and LBP on gait. The aim of our study was to quantify the gait pattern of obese subjects with and without LBP and normal-mass controls by using Gait Analysis (GA), in order to investigate the cumulative effects of obesity and LBP on gait.

**Methods:**

Eight obese females with chronic LBP (OLG; age: 40.5 ± 10.1 years; BMI: 42.39 ± 5.47 Kg/m^2^), 10 obese females (OG; age: 33.6 ± 5.2 years; BMI: 39.26 ± 2.39 Kg/m^2^) and 10 healthy female subjects (CG; age: 33.4 ± 9.6 years; BMI: 22.8 ± 3.2 Kg/m^2^), were enrolled in this study and assessed with video recording and GA.

**Results and Discussion:**

OLG showed longer stance duration and shorter step length when compared to OG and CG. They also had a low pelvis and hip ROM on the frontal plane, a low knee flexion in the swing phase and knee range of motion, a low dorsiflexion in stance and swing as compared to OG. No statistically significant differences were found in ankle power generation at push-off between OLG and OG, which appeared lower if compared to CG. At hip level, both OLG and OG exhibited high power generation levels during stance, with OLG showing the highest values.

**Conclusions:**

Our results demonstrated that the association of obesity and LBP affects more the gait pattern than obesity alone. OLG were in fact characterised by an altered knee and ankle strategy during gait as compared to OG and CG. These elements may help optimizing rehabilitation planning and treatment in these patients.

## Background

Obesity is recognised as a major public health problem in industrialized countries and it is often associated with various musculoskeletal disorders including low back pain (LBP) [1-3]. Some studies have shown a correlation between obesity and functional impairment of the spine secondary to weakness and stiffness of the lumbar muscles, leading to LBP and disability [4-6]. Low flexibility of the spine and increased dorsal stiffness have also been reported in obese subjects [7-9]. Quantitative evidence exists that obesity *per se *affects standing up [10,11], walking [12-14] and running [15,16]. The gait pattern in obese adult males appears generally similar to the one in their lean counterparts, but some of the temporal and angular components have been shown to be different [17]. Excess of mass in fact imposes abnormal mechanics on body movements [18,19]. Body shape is influenced by the excess of mass [20-22], which can hinder the joints' physiological range of motion and enhance the risk of musculoskeletal overload [18].

In addition, other segments' masses, relative muscle strengths, the internal need to optimize gait mechanics for other factors, such as decreased loading on the hip or knee, or optimizing to decrease reliance on quadriceps act as important factors in influencing gait [17,23].

It has been suggested that a neuromuscular modulation may occur in morbidly obese subjects during walking to increase ankle muscle function and power and plantar flexion torque [16].

Gait in obese subjects has been quantitatively studied: they generally show a normal pattern with some differences in the temporal and angular components, for which the excessive adipose tissue in the thighs might mainly account [15,17,24]. In the literature, the analysis of gait pattern in subjects affected by LBP has received scant attention. Few studies have used different techniques (clinical assessment, accelerometers, 3D movement analysis) and experimental conditions (activities of daily living, treadmill and ground walking) [25-29]. To our knowledge only two quantitative studies investigated the capacity of normal-mass subjects with LBP vs. those without to adapt their gait pattern to different velocities during treadmill walking [27,28]. These analyses were conducted in terms of trunk and pelvis rotations and erector spinae muscular activity in order to evaluate the trunk-pelvis coordination. They found that LBP patients had difficulties in modulating trunk-pelvis coordination, especially on the transversal plane, and erector spinae activity following velocity perturbations. In another study, Spenkelink et al. [26] investigated differences between LBP patients and healthy subjects in daily living activities and the day-to-day variability using an ambulant monitoring system based on accelerometers. In this study, LBP patients showed lower activity levels with lower gait velocity and time spent in standing position and greater time spent resting than the control group.

Despite being frequently associated, the effects of LBP and obesity on biomechanical gait parameters have been investigated separately. None of them has investigated the combined effects of obesity and LBP and the quantitative differences in gait strategy in obese with LBP as compared to those without LBP. We have previously shown [9] that in those subjects LBP maybe a consequence of obesity itself and it could possibly be prevented by weight loss and specific spine exercises. Our hypothesis was that LBP combined with obesity might worsen the gait pattern of obese subjects. Bearing in mind that walking is a key component of comprehensive rehabilitation in obesity, verifying our hypothesis would reinforce the need for specific and preventive spine exercises and weight loss programmes.

A deeper understanding of the causes of their gait abnormalities, and ultimately of their motor disability, may help optimizing rehabilitation planning and treatment. 3-D Gait Analysis (GA) is nowadays the most accurate tool to investigate the gait pattern. From a clinical perspective, measuring the joint angular displacement, reactions, moments and powers provides insight into the 'how' (kinematics) and the 'why' (kinetics) of the movement observed. No studies up to now have addressed this issue of defining quantitative differences in gait strategy in patients with obesity and LBP.

The aim of our study was therefore to quantify the gait pattern of obese subjects with and without LBP and normal-mass controls by using GA, in order to investigate the combined effects of obesity and LBP on gait.

## Materials and methods

### Participants

We included in the study 8 obese females (BMI ≥ 35 Kg/m^2^) with chronic LBP (OLG group). According to the Italian Guidelines on LBP [30], chronic LBP was defined as lumbar pain with no evidence of specific origin lasting more than 3 months. Our subjects had a chronic non-specific pain and were not under any medication at the time of the experiment. We excluded subjects with secondary LBP, sciatica, osteoporosis, osteoarthritis, rheumatologic, metabolic or hematologic abnormality with potential to affect lower limb function and neurological diseases precluding physical exercise, cardio-respiratory conditions (diagnosed after treadmill stress tests), acute illnesses. Two control groups were recruited for this study: the first group included 10 matched obese females without LBP (OG) and the second one included 10 age-matched normal-mass females (CG). OG and CG reported no history of LBP during the last six months.

All the obese participants were admitted to our Unit for multidisciplinary rehabilitation program for obesity. We considered three groups of female subjects in order to avoid gender differences and because the prevalence of LBP is greater in women than in men [31].

All the subjects were able to understand and complete the test and walk independently without aids. They did not report pain while walking.

The study was approved by the Ethics Research Committee of our Institute and written informed consent was obtained from the patients.

### Methods

Physical evaluation included: height (expressed in meters), waist circumference, and mass (expressed in Kg) determination. Moreover, BMI (kg/m^2^) was calculated. Waist circumference was measured with a plastic tape midway between the lowest rib and iliac crest with the subject standing at the end of gentle expiration and expressed in cm [32]. All the details of the three groups are reported in Table 2.

The clinical examination included palpation of the paravertebral muscles, straight leg raising test for sciatic nerve impingement, tendon reflexes of the lower limbs. Subjective perception of pain was evaluated with a Visual Analogue Scale (0-10).

All subjects were randomly evaluated at the Movement Analysis Lab of our Institute using an optoelectronic system with 6 cameras (460 VICON, Oxford Metrics Ltd., Oxford, UK) with a sampling rate of 100 Hz, and two force platforms (Kistler, CH).

The optoelectronic system performs a real time processing of images from 6 fixed infra-red cameras to extract the reflectance of passive markers (with a diameter of 15 mm) which are positioned on specific anatomical landmarks of the subject. Prior to testing, the system was calibrated to assure accuracy and to allow the computation of each marker's 3D coordinates using a right-handed coordinate system and standing calibrations were performed on all subjects prior to data collection. The accuracy (Mean Residual) was 0.87 mm and the static reproducibility was 0.81%.

To evaluate the kinematics of each body segment, passive markers were positioned on the subject's body, as described by Davis [33]. Particular attention was taken for placement of the markers, especially at the pelvis (anterior superior iliac spine and posterior superior iliac spine). After placement of the markers, subjects were asked to walk barefoot at their own natural pace (self-selected speed) along a walkway with embedded force platforms at the mid-point. At least six acquisitions were collected for each patient in order to guarantee reproducibility of the results.

### Data analysis

The 3D orientation of body segments of interest (pelvis, left and right thighs, left and right shanks, both feet) was obtained by tracking trajectories according to a common commercially available kinematic model (Plug-In-Gait, Vicon Peak, Oxford, UK). All graphs obtained from GA were normalized as % of gait cycle and kinetic data were normalized for individual body mass.

From the analysis of these graphs we identified some parameters (time/distance parameters, joint angle values at specific instances of the gait cycle, peak values in joint power graphs). Among all parameters calculated, we selected the most reliable and consistent for analyses in individuals characterised by obesity [14,17,24,34] (Table 1):

**Table 1:** Gait parameters and descriptors.

Gait Parameter	Description
*Spatio-temporal parameters*	

% stance (%gait cycle)	% of gait cycle that begins with initial contact and ends at toe-off of the same limb;

Double stance phase (% gait cycle)	% of gait cycle when both feet are on the ground

Mean velocity (1/s)	mean velocity of progression normalised for individual height

Step length	longitudinal distance from one foot strike to the next one, normalized to subject's height

Cadence (step/min)	Number of step for

*Kinematics (degrees)*	

HFE-ROM	the range of motion at hip joint on the sagittal plane during the gait cycle, calculated as the difference between the maximum and minimum values of the plot

HAA-ROM	the range of motion at hip joint on the frontal plane (Hip Ab-Adduction graph) during the gait cycle, calculated as the difference between the maximum and minimum values of the plot

KIC	value of Knee Flexion-Extension angle (knee position on sagittal plane) at initial contact, representing the position of knee joint at the beginning of gait cycle

KmSt	minimum of knee flexion (knee position on sagittal plane) in mid-stance, representing the extension ability of knee during this phase of gait cycle

KMSw	peak of knee flexion (knee position on sagittal plane) in swing phase, representing the flexion ability of knee joint during this phase of gait cycle

KFE-ROM	the range of motion at knee joint (on the sagittal plane) during the gait cycle, calculated as the difference between the maximum and minimum values of the plot

AIC	value of the ankle joint angle (on sagittal plane) at the initial contact, representing the position of knee joint at the beginning of gait cycle

AMSt	peak of ankle dorsiflexion (on sagittal plane) during stance phase, representing the dorsiflexion ability of ankle joint during this phase of gait cycle

AmSt	minimum value of the ankle joint angle (on sagittal plane) in stance phase, representing the plantarflexion ability of ankle joint at toe-off

AMSw	peak of ankle dorsiflexion (on sagittal plane) during swing phase, representing the dorsiflexion ability of ankle joint in this phase of gait cycle

ADP-ROM	the range of motion at ankle joint (on the sagittal plane) during the stance phase, calculated as the difference between the maximum and minimum values of the plot in this phase of gait cycle

*Kinetics (W/Kg)*	

APMax	the maximum value of generated ankle power during terminal stance, representing the push-off ability of the foot during walking

APmin	minimum value of absorbed ankle power in early stance and mid-stance, when muscle is contracting eccentrically and absorbing energy

HPMax	the maximum value of generated hip power during midstance

Spatio-temporal parameters:

- % stance (as % of the gait cycle);

- Double stance phase (as % of the gait cycle);

- mean velocity, normalised for individual height (1/s);

- step length, normalised for individual height;

- cadence: number of steps in a time unit (steps/min).

Kinematics:

- the values of angle of ankle (AIC index) and knee (KIC index) at the Initial Contact (IC);

- the values of ankle dorsiflexion peak during stance (AMSt index) and swing phase (AMSw index) and the peak of knee flexion (KMSw index) during swing;

- the values of peak of ankle plantarflexion in stance (AmSt index) and of knee extension (KmSt index) during the gait cycle;

- the hip ROM on the coronal (HAA-ROM index) and sagittal (HFE-ROM) plane; the knee ROM (KFE-ROM index) on the sagittal plane; the ankle ROM on the sagittal plane during stance (ADP-ROM index).

Kinetics:

- the maximum value of absorbed power during loading response and mid-stance (respectively from 0% to 10% and from 10% to 30% of the gait cycle); APmin index, W/Kg), representing the ability to absorb the impact of the foot to the ground;

- the maximum ankle power during terminal stance (from 30% to 50% of the gait cycle) (APMax index, W/Kg), representing the push-off capacity during walking and related to the forward propulsive power during gait;

- the maximum value of the generated hip power in midstance (from 10% to 30% of the gait cycle) (maximum value of positive hip power in the first phase of stance; HPMax index, W/Kg).

### Statistical analysis

All the previously defined parameters were computed bilaterally for each participant and the mean and standard deviation values of all indexes were calculated for each group. Data of the right and the left side were compared using Wilcoxon signed rank test.

Kolomogorov-Smirnov tests were used to verify if the parameters were normally distributed; the parameters were not normally distributed, so we used analysis of variance for nonparametric (Kruskall-Wallis) data followed by a post hoc range analysis. The post hoc range analysis was performed using the Mann-Whitney U-test. P-values less than 0.05 were considered significant.

Further the differences between OLG and OG were estimated using the Cohen effect size (d') [35]. Responsiveness is considered to be "trivial" for d' < 0.20, "small" for 0.20 ≤ d' < 0.50, "moderate" for 0.50 ≤ d' < 0.80, and "large" for d' ≥ 0.80.

## Results

Age did not significantly differ among groups. BMI, mass and height were similar in OLG and OG but significantly different from CG (Table 2). OLG and OG showed larger waist circumference than CG, with OLG significantly larger than OG. The BMI between the OLG and OG groups was similar but the waist measurement was significantly greater in the OLG versus the OG group. The VAS score was 5.9 ± 1.8 for OLG and it was 0 for OG and CG.

**Table 2:** Clinical characteristics of the studied groups.

	OLG	OG	CG
Age (years)	40.5 ± 10.1	33.6 ± 5.2	35.8 ± 9.3

Height (m)	1.59 ± 0.05+	1.58 ± 0.03+	1.69 ± 0.07

Weight (Kg)	107.1 ± 16.9+	98.3 ± 5.3+	64.5 ± 8.3

BMI (Kg/m^2^)	42.4 ± 5.5+	39.3 ± 2.4+	22.4 ± 4.6

Waist circumference (cm)	124.8 ± 7.8*+	107.3 ± 9.2+	64.2 ± 10.8

Data are expressed as mean (standard deviation).

* = p < 0.05, OLG compared to OG; + = p < 0.05, OLG and OG compared to CG

First, a comparison between all the parameters (spatio-temporal, kinematic and kinetic) of the right and left limb was made for all participants. No significant differences were found between the two limbs, indicating a symmetric gait pattern. Subsequently, data from both sides were pooled.

In Table 3 I, the mean values and standard deviations of the spatio-temporal, kinematic and kinetic indexes for the three groups are reported.

**Table 3:** Spatio-temporal and kinematic parameters of the study groups.

	OLG	OG	CG	d'
*Spatio-temporal parameters*				

%stance (% gait cycle)	63.44 (1.24)* +	61.99 (1.00)	59.45 (1.45)	1.30

Double stance phase (% gait cycle)	26.54 (2.44)+	25.01 (2.14)+	23.60 (3.65)	0.67

Step length	0.34 (0.06)*+	0.38 (0.06)+	0.88 (0.21)	1.08

Cadence (step/min)	113.67 (5.99)	111.29 (8.28)	111.80 (4.80)	0.32

Velocity (1/s)	0.69 (0.07)+	0.71 (0.09)+	0.79 (0.07)	0.27

*Hip joint (°)*				

HFE-ROM	42.99 (5.84)	42.89 (4.35)	43.52 (4.76)	0.02

HAA-ROM	15.12 (2.60)*+	18.78 (1.81)+	10.71 (3.06)	1.66

*Knee joint (°)*				

KIC	3.27 (3.87)	4.23 (3.89)	4.06 (6.63)	0.25

KmSt	-2.63 (3.94)	-0.13 (4.23)	0.12 (3.82)	0.61

KMSw	52.23 (6.72)* +	58.12 (3.22)	59.01 (6.18)	1.19

KFE-ROM	55.26 (6.45)*+	58.16 (5.41)	60.28 (6.31)	0.55

*Ankle joint (°)*				

AIC	-3.51 (3.03)	1.28 (2.15)	1.81 (4.87)	1.85

AMSt	10.51 (1.17)*+	14.52 (2.98)	13.04 (5.16)	1.45

AmSt	-14.32 (4.81)+	-17.08 (5.06)+	-11.74 (9.40)	0.54

ADP-ROM	27.95 (4.21)	30.55 (5.89)	22.72 (6.56)	0.59

AMSw	2.81 (2.54)* +	5.05 (1.85)	5.63 (4.93)	1.02

*Hip Power (W/Kg)*				

HPMax	2.41 (1.73)* +	1.05 (0.54)+	0.67 (0.37)	1.20

*Ankle Power (W/Kg)*				

APmin	-1.05 (0.21)+	-0.98 (0.19)+	-0.50 (0.29)	0.37

APMax	2.84 (0.64)+	3.05 (0.61)+	3.75 (0.86)	0.34

Data are expressed as mean (standard deviation).

* = p < 0.05, OLG compared to OG; + = p < 0.05, OLG and OG compared to CG

(ROM: Range Of Motion; PT: Pelvic Tilt; PO: Pelvic Obliquity; HIC: Hip at IC; HFE: Hip Flex-Extension; HAA: Hip Ab-Adduction; KIC: Knee at IC; KFE: Knee Flex-Extension; AIC: Ankle at IC; ADP: Ankle Dorsi-Plantarflexion; AP: Ankle Power; HP: Hip Power; IC: Initial Contact; St: Stance; Sw: Swing; M and Max: maximum value; m and min: minimum value). Cohen effect Size d' is calculated to estimate the difference between OLG and OG.

As for the spatio-temporal parameters, OLG were characterised by significantly longer stance duration and by shorter step length than OG and CG (p < 0.05). Double stance phase duration was longer and velocity of progression was less in OLG and OG if compared to CG, while cadence was similar in the three groups.

The hip Range Of Motion (HFE-ROM index) of the two pathological groups was similar to CG. As for hip ab-adduction, both OLG and OG were characterised by an increased hip movement in the frontal plane as compared to CG, with OG showing the highest values than OLG.

OLG and OG showed a quasi-physiological knee motion during the whole stance (KIC and KmSt indices) with no significant differences. During the swing phase, OLG showed lower knee flexion and KFE-ROM index as compared to OG and CG (p < 0.05). The analysis of ankle kinematics showed a lower dorsiflexion during the whole gait cycle (AMSt and AMSw indices) in OLG as compared to OG and CG (p < 0.05). Both OLG and OG were characterised by an increased plantar flexion at toe-off (AmSt index) than CG and a functional ROM (ADP-ROM index).

The analysis of kinetic parameters showed that the maximum ankle power (APMax index) was lower in OLG and OG if compared to CG and the peak of absorbed ankle power (APmin index) during the impact on the ground was significantly greater in OLG and OG than CG (p < 0.05). OLG and OG presented greater values of hip power generation in the first part of stance (HPMax index) as compared to CG, with OLG showing the highest values (p < 0.05) than OG. All the results were confirmed using the exact probabilities for small samples. In addition, all the significant parameters in the comparison between OLG and OG presented a large effect size (Cohen d' > 0.80).

## Discussion

In this study we aimed to compare quantitatively the gait pattern of obese subjects with and without LBP by using GA. Our results seem to stratify the gait patterns of obese with LBP from those without and from normal-mass subjects. The presence of LBP induces some further alterations of the gait pattern compared to obese subjects without LBP (OG), who already yield some distinct biomechanical features from normal-mass controls. The gait pattern in OG appears somehow in between those observed in OLG and CG. The coexistence of obesity and LBP seems therefore to affect gait more severely than obesity alone. OLG were characterised by a reduced stability during gait, as assessed by prolonged stance duration and lower velocity and step length if compared to OG and healthy subjects, and a less physiological knee and ankle strategy. It can be speculated that pain-relief may represent the major underlying cause of this strategy: ankle dorsiflexion and knee flexion can potentially provoke traction on the sciatic nerve, inducing subjects to cautiously limit their range of motion. It has to be reminded, however, that we had excluded subjects affected by clinically sound sciatic pain.

In terms of kinetics, we can observe that the low ankle power generation (APMax index) which characterised OLG group resulted in greater power at hip level (HPMax index). This means that patients did not generate much walking power from their ankle plantar flexors and as a result, they had to obtain additional power from their hips. This strategy is less accentuated in OG as they present greater hip power generation than CG, but lower than OLG, and ankle power generation close to CG; these results are in agreement with previous study [14].

The abnormal spatio-temporal parameters have been associated to an underlying instability in the obese, with a greater period of double support associated to a slower gait velocity in order to guarantee the maintenance of dynamic balance [36]. OG displayed greater movement of pelvis and hip on the frontal plane and a dorsiflexion position in stance phase. While the abduction of the weight-bearing hip may be connected to a larger thigh girth, the dorsiflexed ankle position during stance phase was supposed to reflect low hip flexion in midstance (HmSt index) coupled with a shorter step length [36].

Despite the consistent finding that subjects affected by LBP walk more slowly than pain-free individuals and with abnormal trunk-pelvis coordination [27-29], to our knowledge, no previous quantitative analyses of the biomechanical strategy of lower limb in obese subjects with LBP during gait have been performed.

We decided to focus our attention on walking, as it represents the most common modality of physical activity, significantly contributing to mass reduction programs for obese subjects [37,38].

Walking at a constant intensity for a prolonged lapse of time is a useful and frequently used strategy for body mass reduction in obese patients because it is a convenient type of physical activity that can be used to expend a significant amount of metabolic energy [39]. Therefore walking abnormalities should be taken into account to avoid overload and possible musculoskeletal problems leading to prolonged rehabilitation phase. Knowledge of biomechanical alterations induced by LBP can help tailoring specific treatment to recover gait pattern. The effect of obesity and LBP on gait could in fact generate a vicious circle hampering the rehabilitation process. In addition to spine flexibility and strengthening, range of motion exercises at knee level, strengthening of distal (ankle dorsiflexor) and proximal (knee flexor) muscles should be part of the rehabilitation program in obese patients with LBP.

The main limitation of this study is the small sample size resulting in limited strength of the clinical and statistical findings. Our analysis was limited only to women with a moderate degree of LBP, as evidenced by the VAS score. No standardised flexibility tests were performed. Further studies should be conducted in a larger sample size, in individuals with a more severe LBP and also providing clinical and functional scores. The differences identified in this study were not so large, which may be related to the moderate degree of LBP reported by the patients. However this represents a first attempt to quantify, from a biomechanical point of view, the functional limitation during gait in a group of obese subjects with LBP.

## Conclusions

In conclusion, this study shows a different motor strategy during gait among OLG, OG and CG. From a clinical point of view, our results suggest that rehabilitation program should include specific treatments to improve gait pattern in obese patients with and without LBP. Parallel to weight loss, gait retraining and selective muscle strengthening with attention to ankle, hip and pelvis strategies appears therefore crucial to prevent possible musculoskeletal disorders, improve the gait pattern and the ability to sustain this key aerobic activity for managing mass reduction programs.

## Competing interests

The authors declare that they have no competing interests.

## Authors' contributions

All authors read and approved the final manuscript

VC made substantial contributions to analysis and interpretation of data and was involved in drafting the manuscript

LV made substantial contributions to data acquisition, elaboration and interpretation

MG made contribution to conception, design and interpretation of data, revising the manuscript critically and gave the final approval of the manuscript

FZ made contribution to interpretation of data, revising the manuscript critically

SN made contribution to interpretation of data, revising the manuscript critically

PC made contribution to conception, design and interpretation of data, revising the manuscript critically and gave the final approval of the manuscript
